# Dual binding mode of antithyroid drug methimazole to mammalian heme peroxidases – structural determination of the lactoperoxidase–methimazole complex at 1.97 Å resolution

**DOI:** 10.1002/2211-5463.12051

**Published:** 2016-06-14

**Authors:** Rashmi Prabha Singh, Avinash Singh, Harsh Vardhan Sirohi, Amit Kumar Singh, Punit Kaur, Sujata Sharma, Tej P. Singh

**Affiliations:** ^1^ Department of BiophysicsAll India Institute of Medical SciencesNew DelhiIndia

**Keywords:** antithyroid drug, crystal structure, distal heme pocket, lactoperoxidase, mammalian heme peroxidases, methimazole

## Abstract

Lactoperoxidase (LPO, EC 1.11.1.7) is a member of the mammalian heme peroxidase family which also includes thyroid peroxidase (TPO). These two enzymes have a sequence homology of 76%. The structure of LPO is known but not that of TPO. In order to determine the mode of binding of antithyroid drugs to thyroid peroxidase, we have determined the crystal structure of LPO complexed with an antithyroid drug, methimazole (MMZ) at 1.97 Å resolution. LPO was isolated from caprine colostrum, purified to homogeneity and crystallized with 20% poly(ethylene glycol)‐3350. Crystals of LPO were soaked in a reservoir solution containing MMZ. The structure determination showed the presence of two crystallographically independent molecules in the asymmetric unit. Both molecules contained one molecule of MMZ, but with different orientations. MMZ was held tightly between the heme moiety on one side and the hydrophobic parts of the side chains of Arg255, Glu258, and Leu262 on the opposite side. The back of the cleft contained the side chains of Gln105 and His109 which also interacted with MMZ. In both orientations, MMZ had identical buried areas and formed a similar number of interactions. It appears that the molecules of MMZ can enter the substrate‐binding channel of LPO in two opposite orientations. But once they reach the distal heme pocket, their orientations are frozen due to equally tight packing of MMZ in both orientations. This is a novel example of an inhibitor binding to an enzyme with two orientations at the same site with nearly equal occupancies.

**Abbreviations**
ABTS: 2,2′‐azino‐bis(3‐ethylbenzothiazoline‐6‐sulphonic acid
EPO: eosinophil peroxidase
LPO: lactoperoxidase
MMZ: methimazole
MPO: myeloperoxidase
PEG: polyethylene glycol
TPO: thyroid peroxidase

The family of mammalian peroxidases with a covalently linked prosthetic heme group consists of four members including myeloperoxidase (MPO), lactoperoxidase (LPO), eosinophil peroxidase (EPO), and thyroid peroxidase (TPO). These enzymes use hydrogen peroxide (H_2_O_2_) as the electron accepter to catalyze numerous oxidative reactions. The preferred substrates for MPO, LPO, EPO, and TPO are chloride 1, 2, thiocyanate 3, 4, bromide 5, 6, and thyroglobin 7, 8, respectively, although all the four enzymes have been shown to act on all halides 9, 10, 11, 12 as well as on various organic aromatic compounds 13, 14, 15. The crystal structures of MPO in unbound states 16, 17 as well as in a bound state 18 and LPO in an unbound state 19 as well as in bound states 20, 21, 22, 23, 24, 25, 26, 27 are available. These structures showed that the shapes and sizes of the substrate‐binding clefts on the distal heme side in these enzymes are similar. Unfortunately, crystal structures of TPO and EPO are not yet known. However, because of high homologies in the amino acid sequences of MPO, LPO, EPO, and TPO as well as their very similar functional properties, the cavities which bind to the substrates of EPO and TPO are speculated to be similar to that of MPO and LPO. Hence, the available structural information of LPO and MPO can be exploited for designing potential antithyroid drugs which could bind specifically to TPO.

It has been shown that TPO is involved in the iodination and coupling of tyrosyl residue in thyroglobulin that results in the formation of thyroxin and triidothyronine 28, 29. It is also well known that overproduction of thyroid hormones leads to hyperthyroidism. The antithyroid drug, methimazole (MMZ) (1‐methyl‐2‐mercaptoimidazole) has been extensively used in clinical practice for controlling the hyperthyroidism 30, 31, 32. The effect of MMZ on LPO‐catalyzed oxidation has also been studied and the value of the IC_50_ for the oxidation of 2,2′‐azino‐bis‐3‐ethyl‐benzothiazoline‐6‐sulphonic acid (ABTS) which is catalyzed by LPO has been estimated to be ~ 7.0 μm 33. However, the mode of binding and mechanism of action of MMZ to TPO/LPO are not yet known. LPO has also been shown to play a similar role in the process of iodination 34 indicating a similar catalytic action of LPO as that of TPO 35. On the other hand, LPO and TPO share an overall sequence homology of 76% while the amino acid residues of the substrate‐binding cleft on the distal heme side are fully conserved (Fig. 1). Because of these various similarities between LPO and TPO, the binding and structural studies of the complex of LPO with antithyroid drug MMZ (Fig. 2A) may provide valuable insights into the mode of binding of MMZ to TPO. Therefore, we have determined the crystal structure of the complex of LPO with MMZ.

**Figure 1:**
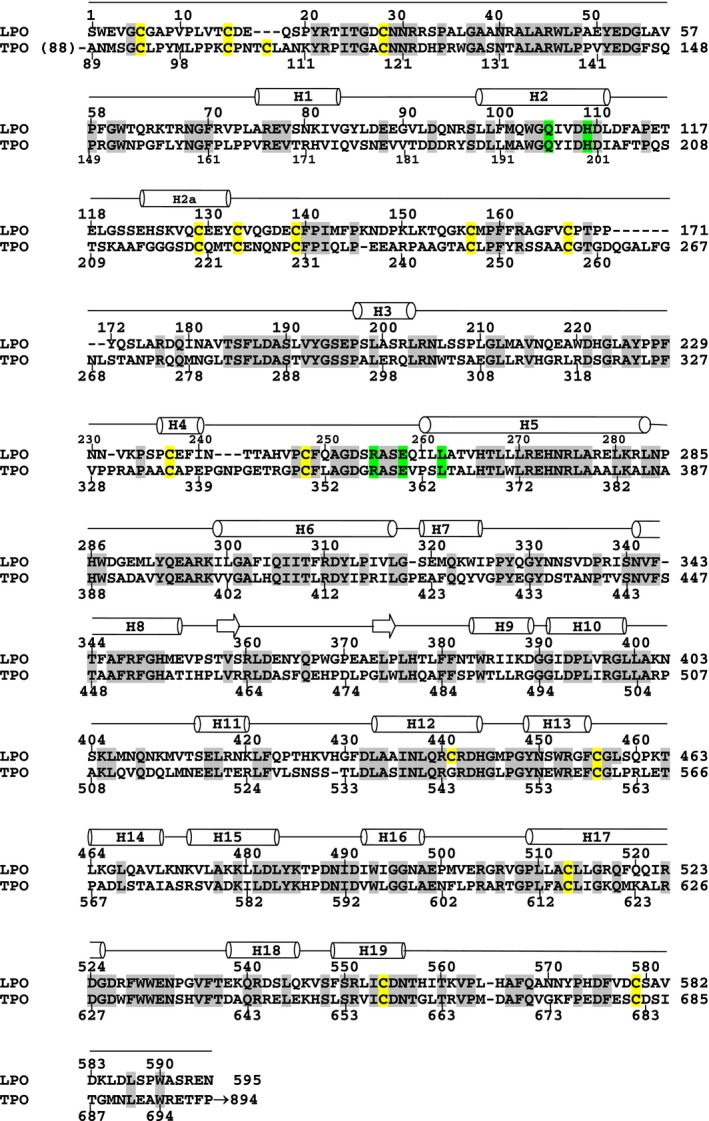
Sequence alignment of LPO (residues 1–595) and TPO (residues 89–699). The identical residues are highlighted in gray while conserved residues on the distal heme side are highlighted in green. Cysteine residues are highlighted in yellow. Secondary structure elements, α‐helices (cylinders) and β‐strands (arrows) are indicated above the sequences.

**Figure 2:**
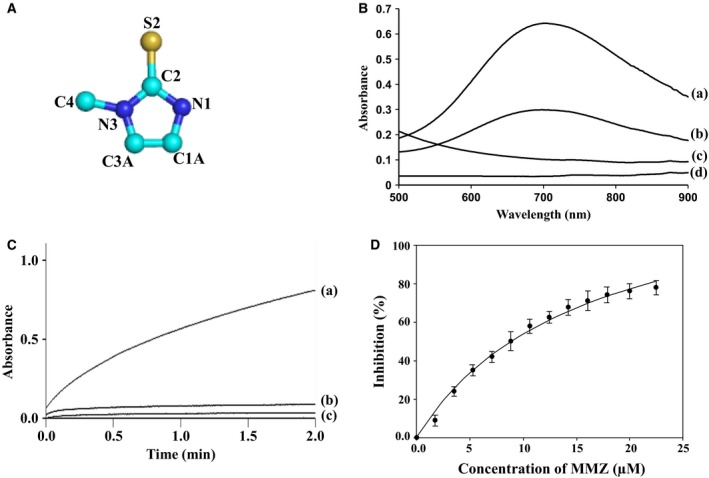
(A) Chemical structure of methimazole (MMZ), (1‐Methyl‐2 mercaptoimidazole) with numbering scheme. (B) Absorption spectra for the product formed between (a) reagents, potassium ferricyanide and ferric chloride with MMZ, (b) reagents and MMZ obtained from crystals, (c) reagents in buffer and (d) MMZ in buffer. (C) The activity curves for LPO as estimated using (a) purified LPO, (b) crystals of the complex of LPO with MMZ and (c) LPO and MMZ in 1 : 1 molar ratio. (D) For determining the value of IC_50,_ a plot for estimating the absorptions at 412 nm for the fixed concentration of LPO with varying concentrations of MMZ.

## Results

### Detection of MMZ in crystals

As seen from Fig. 2B, the absorption spectra were recorded in the wavelength range of 500–900 nm for the products from four independent experiments, (a) reaction reagents, potassium ferricyanide and ferric chloride and MMZ, (b) reaction reagents and MMZ which were obtained from crystals of the complex of LPO with MMZ, (c) reaction reagents alone in buffer, and (d) MMZ alone in buffer. It showed that the absorption maxima in curves (a) and (b) occurred at a wavelength of 713 nm corresponding to the product of the reaction between reagents and MMZ. The other two curves represented by (c) and (d) corresponding to only reagent and only MMZ, respectively, did not have absorption maxima and hence indicated the absence of a similar product with blue color. The difference in the peak heights of curves (a) and (b) may be due to low concentration of MMZ in the experiment with a sample from the crystals. These observations clearly showed that MMZ was present in the crystals which were used for the data collection.

### Activity/Inhibition of LPO by MMZ

The activity curves obtained for the (a) purified samples of LPO, (b) samples obtained by crushing the washed crystals of the complex of LPO with MMZ, and (c) samples of LPO mixed with MMZ at 1 : 1 molar ratio are shown in Fig. 2C. These clearly showed that the activity of LPO was considerably reduced in the presence of MMZ. As indicated by curve (b) in Fig. 2B, the activity of LPO was almost absent in the crystals. It was nearly similar to that of LPO in solution at 1 : 1 molar ratio with MMZ. The slight difference in curves (b) and (c) may be due to low occupancy of MMZ in the crystals. However, in both cases, it showed that the activity of LPO was inhibited by MMZ apparently by blocking the binding of H_2_O_2_ to heme iron.

As seen from Fig. 2D, 50% inhibition of LPO activity was achieved at 8.84 μm concentration of MMZ indicating a significant affinity for the binding of MMZ to LPO. This value is slightly more than the value reported in literature 33. However, there is no ambiguity about the inhibition of the function of LPO by MMZ.

### Analysis of the structure of the complex of LPO with MMZ

**Figure 3:**
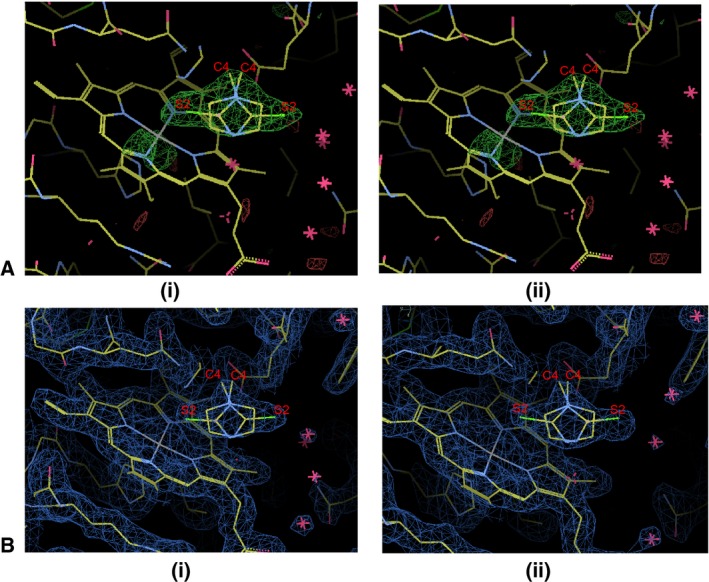
(A) Showing fittings of two orientations in the OMIT (Fo–Fc) electron density map for MMZ at 3 σ cutoff in (i) molecule A and (ii) molecule B. (B) Showing molecules of MMZ in the final (2F_o_ − F_c_) electron density map at 1 sigma cutoff in (i) Molecule A and (ii) Molecule B.

**Figure 4:**
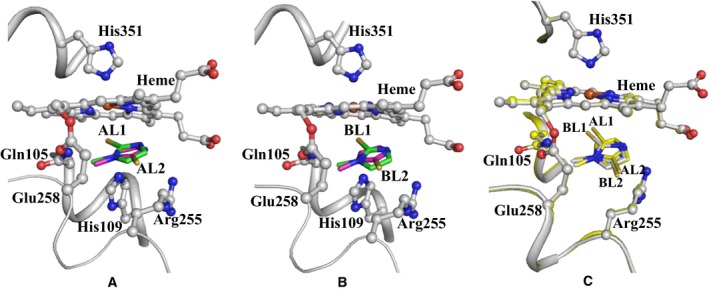
The views of MMZ are shown in the clefts in (A) molecule A and (B) molecule B. The surrounding residues are also shown. (C) The superimposition of molecules of MMZ in the cleft on the distal heme side are shown.

**Figure 5:**
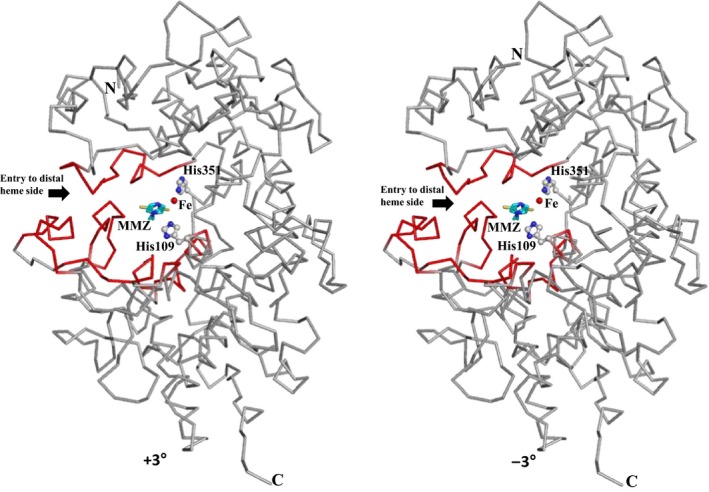
Stereoview of molecule A of LPO with a bound MMZ molecule at the substrate‐binding site on the distal heme side. The entry to substrate‐binding channel is indicated by an arrow. The positions of iron atom, His109 and His351 are shown. For the clarity of the position of MMZ, heme moiety is not shown.

**Figure 6:**
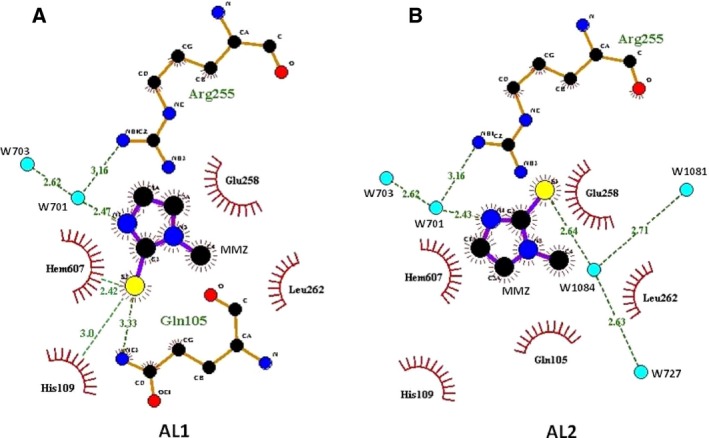
The interactions between LPO and MMZ using program Ligplot+ 50 in (A) orientation AL1 and (B) orientation AL2. The hydrogen bonds are indicated by dashed lines while van der Waals contacts are indicated by arcs. The contact between heme iron and sulfur AL1 is indicated by a continuous line. Such a contact is not there with AL2 because of an away position of sulfur atom.

The final structure of the LPO complexed with MMZ was refined and the values of R_cryst_ and R_free_ factors converged to 0.177 and 0.226, respectively (Table 1). The structure contains two crystallographically independent molecules, A and B, in the asymmetric unit. The structures of both the molecules were identical with an root mean squares shift of 0.4 Å for the C^α^ atoms. The MMZ omit (F_o_ − F_c_) (Fig. 3A) and a final (2F_o_ − F_c_) (Fig. 3B) electron density maps clearly showed that in both molecules A and B, MMZ bound to LPO in the substrate‐binding cleft on the distal heme side with two opposite orientations. Both orientations of MMZ were found to have equal occupancies with a similar number of interactions with protein molecules (Table 2). The orientations and positions of MMZ in both protein molecules were identical (Fig. 4). Hereafter, only one molecule of the complex containing LPO and MMZ (Fig. 5) will be used in further discussion. In orientation, AL1, atom S2 of MMZ formed contacts with heme iron, His109N^ε2^, and Gln105N^ε2^ while in orientation, AL2, S2 atom made contacts with water molecule W5′ (W1084) (Table 2). In both orientations, MMZ formed several van der Waals contacts with atoms of neighboring residues in the cleft, which included Gln105, His109, Arg255, Glu258, Leu262 as well as with the atoms of the heme moiety (Fig. 6). The structure has been deposited in the Protein Data Bank with the ID PDB: 5FF1.

**Table 1:** Data collection and refinement statistics

Space group	P2_1_
Unit cell dimensions	
a (Å)	80.3
b (Å)	93.0
c (Å)	81.5
β (°)	89.9
Number of molecules in asymmetric unit	2
Vm (Å3/Da)	2.2
Solvent content (%)	46.0
Resolution range (Å)	40.4–1.97
Total number of measured reflections	156766
Number of unique reflections	78383
Overall completeness of data (%)	93.8 (83.2)
Overall R_sym_ (%)	7.3 (48.3)
I/σ(I)	7.2 (2.1)
R_cryst_ (%)	17.7
R_free_ (%)	22.6
Protein atoms	9509
Water oxygen atoms	775
Carbohydrate atoms	126 (9 NAG)
Ligand atoms in two orientations and half occupancies	28
R.m.s.d. in bond lengths (Å)	0.02
R.m.s.d. in bond angles (°)	1.89
R.m.s.d in torsion angles (°)	15.3
Wilson's B‐factor (Å^2^)	20.9
Mean B‐factor for main‐chain atoms (Å^2^)	30.9
Mean B‐factor for side‐chain atoms and waters (Å^2^)	33.9
Mean B‐factor for all atoms (Å^2^)	32.6
Mean B‐factor for all atoms of MMZ for orientation1 in molecule A and for (orientation 2) (Å^2^)	30.1 (36.6)
Mean B‐factor for all atoms of MMZ for orientation 2 in molecule B and for (orientation 2) (Å^2^)	29.0 (27.8)
Residues in the most allowed regions (%)	88.5
Residues in the additionally allowed regions (%)	11.5
PDB	5FF1

Values in parentheses are for outer resolution shell.

**Table 2:** Distances between the atoms of LPO and MMZ in both orientations up to 4.2 Å

MMZ	LPO	AL1	AL2	BL1	BL2
S2	Fe^3+^	2.42	–	2.66	–
	His109N^ɛ2^	3.01	–	2.86	–
	Gln105N^ɛ2^	3.33	–	2.91	–
	Arg255C^β^	–	3.95	–	4.04
	C^γ^	–	3.72	–	3.52
	C^δ^	–	4.09	–	3.77
	Glu258C^β^	–	3.88	–	3.70
	Glu258C^γ^	–	3.54	–	3.45
	W5′ (1084A)	–	2.64	–	2.78
	(1033B)				
	W7′ (1085A)	–	3.74	–	3.00
	(1042B)				–
C4	His109N^ɛ2^	3.70	–	3.84	–
	His109C^ɛ1^	3.26	–	3.35	–
	Glu258C^β^	3.33	2.79	3.17	2.81
	Glu258C^γ^	3.29	2.84	3.19	2.86
	Glu258C^δ^	3.15	2.90	2.98	2.85
	Leu262C^δ2^	3.96	–	3.84	3.78
C3A	Arg255C^β^	3.52	–	3.58	–
	Arg255C^γ^	3.22	–	3.67	–
	Arg255C^δ^	3.61	–	3.75	–
	Glu258C^β^	3.75	–	3.60	–
	Glu258C^γ^	3.29	–	3.40	–
	Glu258C^δ^	3.15	–	4.02	–
	His109C^ɛ1^	–	3.08	–	2.95
	His109N^ɛ2^	–	2.76	–	2.67
	Gln105N^ɛ2^	–	3.38	–	3.44
C1A	Arg255C^β^	4.08	–	4.07	–
	Arg255C^γ^	3.57	–	3.67	–
	Arg255C^δ^	3.29	–	3.35	–
	His109N^ɛ2^	–	3.15	–	3.03^a^

a N1, C2, and C1A interact with heme atoms.

## Discussion

Methimazole is an antithyroid drug 30, 31, 32. Its primary effect is to interfere with the synthesis of thyroid hormone by inhibiting the action of thyroid peroxidase 36. Initially, it was suggested that MMZ‐like thiocarbamide thyroid inhibitors caused mechanism‐based suicide inactivation of LPO 37, 38. However, recent studies have shown clearly that the inhibition of LPO by MMZ may occur through a competitive coordination of the drug to iron atom which is assisted by other interactions of the drug with protein molecule 39. It is important to note here that the sequences of LPO and TPO have a sequence homology of 76% while the residues on the distal heme side at the periphery of the substrate‐binding cleft are fully conserved (Fig. 1).

The final structure revealed a novel state of binding of molecules of MMZ in the substrate‐binding cleft of LPO with two different orientations. It is important to note that the occupancies of MMZ in both orientations had similar values of ~ 0.5. This showed that MMZ was able to bind to LPO with a similar preference in both orientations. As seen from Fig. 6, in orientation AL1, the sulfur atom S2 of MMZ formed contacts with heme iron, N^ε2^ of Gln105, and N^ε2^ of His109. The methyl group carbon atom C4 of MMZ made many van der Waals contacts with the side chains of His109, Glu258, and Leu262. The ring carbon atom C3A interacted with Arg255 and Glu258 while C1A formed van der Waals contacts with the side‐chain atoms of Arg255. The atoms N1 and C2 made a number of van der Waals contacts with various atoms of the heme moiety. In orientation, AL2, S2 interacts with atoms of the side chains of Arg255 and Glu258 as well as with two water molecules, W5′ (1084 in the PDB) and W7′ (1085 in the PDB). In this orientation, the methyl carbon atom C4 of MMZ formed van der Waals contacts with the side‐chain carbon atoms of Glu258 while atom C3A interacted with the side‐chain atoms of His109 and Gln105. The atom C1A of MMZ formed several van der Waals contacts with His109 as well as with the atoms of heme moiety. The atoms N1 and C2 of MMZ formed van der Waals contacts with several atoms of heme moiety. This showed that MMZ, in both orientations, formed several contacts with the atoms of LPO indicating a similar preference of binding to LPO. It is evident from the buried surface areas that the molecule of MMZ, in both orientations, is fitted tightly between the atoms of LPO, heme moiety, and water molecules.

**Figure 7:**
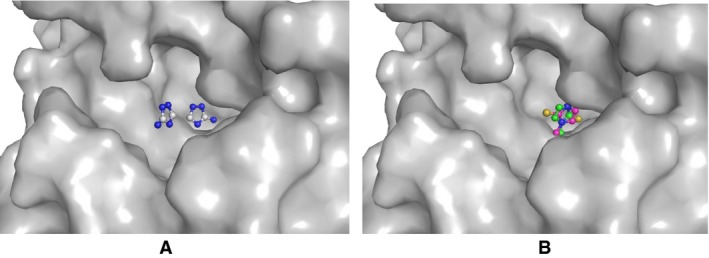
The grasp representation of the substrate‐binding site on the distal heme side in LPO when complexed with (A) amitrole, where two molecules of amitrole were observed, one in the cleft and second outside the cleft. (B) MMZ, where molecules with both orientations are located at the same site inside the cleft.

A comparison of this structure was made with the structure of the complex of LPO with another similar compound, amitrole. 23. The amitrole molecule has a similar structure with a five‐membered ring. The only difference is of an extra attachment of a methyl group in MMZ. In the structure of the complex of LPO with amitrole also, two molecules of amitrole were observed on the distal heme side but in this case, one molecule of amitrole was present inside the pocket on the distal heme side while the second molecule was located in the substrate‐binding channel (Fig. 7A). In contrast, in the complex of LPO with MMZ, MMZ occupied a single position in the pocket on the distal heme side (Fig. 7B). Also the orientations of amitrole and MMZ were slightly different in the pocket. The plane of the five‐membered ring of MMZ was oriented with respect to the plane of the heme moiety at 36° while the corresponding angle for amitrole was 47°. The observed interactions of amitrole with LPO were significantly less than those of MMZ with LPO. This showed that the structure of MMZ with a sulfur atom instead of a nitrogen atom and an extra methyl group were important for enhancing the interactions of MMZ with LPO and hence its potency as an inhibitor.

**Figure 8:**
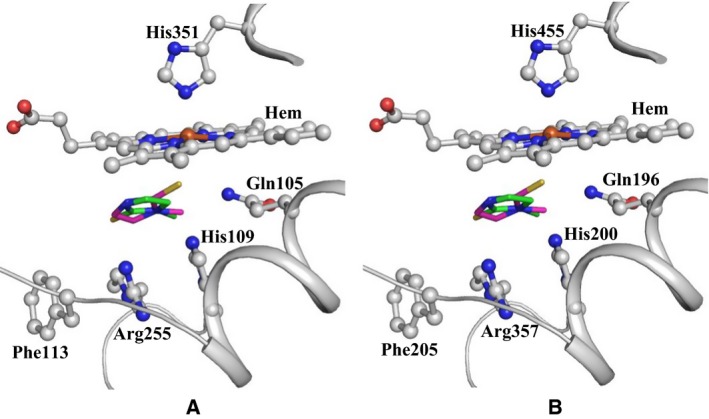
Showing MMZ molecule in the cleft of the substrate‐binding cleft in (A) LPO and (B) TPO.

As the residues surrounding the cleft on the distal heme side in both LPO and TPO are conserved, the similar interactions may occur in the complex of TPO with MMZ as well (Fig. 8). Therefore, MMZ acts as a potent antithyroid drug. The binding of MMZ in two major orientations at the substrate‐binding site on the distal heme side with similar number of interactions shows that MMZ fits into the cleft tightly in these two orientations. In fact, it is so tight in these two orientations that only very low occupancies of its other slightly different orientations could be possible because slight negative densities have been observed at the two opposite sides of the molecule.

To the best of our knowledge, this is the first time that a single compound has been found to bind to the same ligand‐binding site of an enzyme in two opposite orientations with equal occupancies. Based on the structure of the complex of LPO with MMZ, a modification in the structure of MMZ can be suggested for further improving the intermolecular interactions between MMZ and LPO/TPO. In this case, a replacement of the sulfur atom by another atom such as O or N may improve the binding affinity. The synthesis of such compounds is in progress which will be tested for the antithyroid activity. Such an approach may help in preparing new compounds with improved antithyroid activities leading to better therapeutic applications.

## Materials and methods

### LPO purification

The caprine colostrum was obtained from the Indian Veterinary Research Institute, Izatnagar, India. The steps of isolation and purification were modified from the method reported earlier 19 to improve the yield of LPO. In the revised procedure, the buffer50 mm Tris‐HCl, pH8.0 was made in the presence of 2 mm CaCl_2_. The rest of the procedure was as reported earlier 19.

### Crystallization of LPO and soaking with MMZ

About25 mg·mL^−1^ of purified LPO dissolved in 10 mm sodium phosphate buffer at pH 7.0 was used for crystallization, 10 μL protein drops were used for hanging drop vapor diffusion method, with the precipitant as 20% (w/v) PEG‐3350 in 200 mm ammonium nitrate. The green‐colored crystals of LPO were obtained after 7 days. MMZ (Sigma‐Aldrich, St. Louis, MO, USA) was dissolved at a concentration of 50 mg·mL^−1^ in the reservoir solution that additionally contained 20% ethanol. In this solution, LPO crystals were soaked for 52 h.

### Detection of MMZ in the crystals

In order to confirm the presence of MMZ (Fig. 2A) in the crystals, the protocol given by Dong *et al.,* 2009 40 was used. The stocks of standard solutions of 15 mm potassium ferricyanide and 15 mm ferric chloride were prepared. Crystals were transferred to the well containing 10 mm phosphate buffer at pH 7.0. Crystals were washed repeatedly with buffer and then dissolved in the same buffer. This solution was incubated with 1 m NaCl for 1 h and then ultrafiltered using a membrane with a molecular weight cutoff of 1 kDa. The 1 mL of filtrate was mixed with 1 mL of potassium ferricyanide and 1 mL of ferric chloride solutions. After 40 min of incubation, the presence of MMZ in the filtrate was detected by measuring the absorbance in the wavelength range of 500–900 nm against a reagent blank prepared in the same manner but without MMZ (Fig. 2B).

### Estimation of the activity of LPO and its inhibition

The measurement of the activity as well as the inhibition of LPO by MMZ were carried out using (a) purified samples of LPO, (b) samples obtained by crushing the crystals of LPO–MMZ complex, and (c) a solution containing LPO and MMZ at the molar ratio of 1 : 1 The freeze‐dried protein sample was dissolved in a buffer containing 100 mm phosphate buffer atpH 7.0. The LPO activity was determined using the method which has been reported earlier 41. The 3.0 mL of 100 mm 2,2′‐azino‐bis (3‐ethylbenzthiazoline‐sulfonic acid) (ABTS) in 100 mm phosphate buffer, pH 7.0 were mixed with 0.1 mL of 0.5 mg·mL^−1^ protein solution containing 0.1% gelatin to adjust it to zero using spectrophotometer, Lambda 25 (Perkin‐Elmer Life Sciences, Waltham, MA, USA). An amount of 0.1 mL of 3.2 mm hydrogen peroxide was added to the above solution. The absorbance was measured at 412 nm as a function of the oxidized product of ABTS. Similarly, protein samples obtained from the crystals were also tested for the catalytic activity using the procedure described above. For this purpose, the washed crystals were dissolved in 100 mm phosphate buffer at pH 7.0. The activity curves were obtained for the purified samples of LPO, samples containing LPO and MMZ at 1 : 1 molar ratio, and for the samples obtained from the crystals of LPO after soaking the crystals with MMZ (Fig. 2C). Before crushing the crystals for preparing the samples for activity measurements, the crystals were washed three times using the reservoir solution.

### Determination of IC_50_

The inhibition of the activity of LPO by MMZ was carried out and the value of IC_50_ was determined using 5 μm of LPO incubated with varying concentrations of MMZ ranging from 1.0 to 25.0 μm for 20 min at 37 °C. For all the different concentrations of MMZ, the values of absorbance were measured at 412 nm from which % inhibition of LPO activity was extrapolated. A plot has been prepared for the observed relative inhibitions versus the concentrations of MMZ (Fig. 2D). The curve was fitted using the software Sigma plot 8.0 42. All spectroscopic measurements were made using a λ25 Perkin‐Elmer spectrophotometer at 412 nm. Each set of experiment was repeated six times for calculating average values and mean errors.

### Determination of the structure of LPO with MMZ

In order to provide stability to the crystals of the complex of LPO with MMZ, they were immersed in a solution of glycerol (22%) and methanol (20%). The data collection was carried out at a temperature of 100 K. The X‐ray intensity data to 1.97 Å resolution were collected with a MAR CCD‐225 detector (Marresearch, Norderstedt, Germany) using synchrotron beamline, BM14 at the European Synchrotron Radiation Facility (ESRF), Grenoble, France. The program, HKL‐2000 43 was used for processing the data/the crystals belonged to monoclinic space group, P2_1_ with cell dimensions, a = 80.3 Å, b = 93.0 Å, c = 81.5 Å and β = 89.9°. The program POINTLESS clearly showed that the symmetry corresponded to the monoclinic space group, P2_1_ 44. AS indicated by the unit cell dimensions, two molecules of LPO were observed in the asymmetric unit. There were significant differences in the unit cell dimensions of the present structure as compared to those reported earlier 19, 21, 24. Therefore, the structure was determined with molecular replacement method using program PHASER 45. The coordinates of previously determined structure of native LPO 19 were used as a model. The refinement was carried out in the resolution range of 40.4–1.97 Å with program REFMAC 46. The manual model building was carried out using programs O 47 and COOT 48. A difference Fourier (F_o_ − F_c_) electron density map was calculated once the R_cryst_ value became 0.261. The map displayed additional electron densities which did not belong to the protein at > 3.0 σ cutoff. These densities were found in the clefts which bound the substrates in both the molecules on the distal heme sides. The shape of electron densities allowed an appropriate fitting of MMZ molecules with two opposite orientations at the same site (Fig. 3A,B). The coordinates of MMZ molecules in each orientation with a 0.5 occupancy were included in the subsequent cycles of refinement. In orientation AL1/BL1, the sulfur atom was toward the heme iron while in orientation AL2/BL2, it was on the opposite direction. The refinement was carried out with the default restraints for the noncrystallographic symmetry. The default restraints were also used in the refinement of MMZ. The protein chain was adjusted in order to improve the refinement and bring the R_cryst_ factor down and the new (2F_o_ − F_c_) and (F_o_ − F_c_) electron density maps were calculated. A total of 775 water oxygen atoms were observed from these maps. The water molecule, W1 which is observed in the native unbound structure between the position of the substrate and heme iron in the absence of H_2_O_2_ 22 was absent in the present structure. An extra noncharacteristic electron density in the Fo–Fc map at 3 σ cutoff was also observed between the side chain of His351 and heme moiety. At this site, some density has generally been observed in the structures of heme peroxidases (PDB IDs 3N3N, 1ZZH, 3KRQ). It may presumably be due to the very tight positioning of the side chain of His351 from two sides allowing only an up and down movements of the side chain with respect to heme iron. This may result in some residual electron density.

In the subsequent refinement cycles, the coordinates of the water oxygen atoms were included. The final values of R_cryst_ and R_free_ factors were 0.177 and 0.226, respectively. The mean B‐factor for all the atoms in the structure was 32.6 Å^2^. The mean B‐factors for orientations 1 and 2 of MMZ in molecule A of LPO were 30.1 and 36.6 Å^2^, respectively. The corresponding values for MMZ in molecule B of LPO were 29.0 and 27.8 Å^2^, respectively. The B‐factors of individual atoms of MMZ in molecule A for orientation 1 varied from 29.8 to 31.5 Å^2^ while the corresponding values for orientation 2 were 34.9 and 41.5 Å^2^, respectively. The corresponding values for the two orientations in molecule B varied from 27.5 to 30.6 Å^2^ and 25.4 and 31.2 Å^2^, respectively. There were slight negative densities on either sides of MMZ in molecules A and B of LPO which might be due to additional minor conformers of MMZ. The quality of the final model was assessed with PROCHECK 49. A validation report of the final model obtained using wwPDB validation pipeline was also examined. The data collection and refinement statistics are given in Table 1.

## Author contributions

RPS, AS, AKS, SS, and TPS conceived and designed the experiments. RPS, AS, AKS, and TPS performed the experiments. RPS, AS, AKS, HVS, SS, and TPS analyzed the data. RPS, AKS, PK, SS, and TPS contributed reagents/materials/analysis tools. RPS, AS, SS, and TPS wrote the paper.
